# *TEMPRANILLO* is a regulator of juvenility in plants

**DOI:** 10.1038/srep03704

**Published:** 2014-01-15

**Authors:** Tiziana Sgamma, Alison Jackson, Rosario Muleo, Brian Thomas, Andrea Massiah

**Affiliations:** 1 School of Life Sciences, University of Warwick, Gibbet Hill Road, Coventry, West Midlands CV4 7AL, UK; 2 Department of Agriculture, Forests, Nature and Energy, University of Tuscia, Via San Camillo de Lellis snc, Viterbo 01100, Italy

## Abstract

Many plants are incapable of flowering in inductive daylengths during the early juvenile vegetative phase (JVP). *Arabidopsis* mutants with reduced expression of *TEMPRANILLO* (*TEM*), a repressor of *FLOWERING LOCUS T* (*FT*) had a shorter JVP than wild-type plants. Reciprocal changes in mRNA expression of *TEM* and *FT* were observed in both Arabidopsis and antirrhinum, which correlated with the length of the JVP. *FT* expression was induced just prior to the end of the JVP and levels of *TEM1* mRNA declined rapidly at the time when *FT* mRNA levels were shown to increase. *TEM* orthologs were isolated from antirrhinum (*AmTEM*) and olive (*OeTEM*) and were expressed most highly during their juvenile phase. AmTEM functionally complemented AtTEM1 in the *tem1* mutant and over-expression of *AmTEM* prolonged the JVP through repression of *FT* and *CONSTANS* (*CO*). We propose that TEM may have a general role in regulating JVP in herbaceous and woody species.

Flowering time in plants is affected by both developmental and environmental factors. Many plants require a permissive daylength to initiate flowers, sometimes in combination with a particular temperature history. However, in the early stages some plants are incapable of initiating flowering, even when grown under favourable environmental conditions. This is known as the juvenile vegetative phase (JVP), which precedes the adult vegetative phase (AVP), in which reproductive competence is established and the plant can respond to inductive conditions. The progression from the JVP to AVP is a distinct process from the vegetative to reproductive transition.

Vegetative phase change is usually considered as a wider phenomenon in which physiological markers that characterise juvenility have been identified in different species. Attainment of floral competence is the most distinct and consistent manifestation of phase change and hence could be regarded as the most robust indicator of the end of juvenility^1^.

Most plants will eventually initiate flowers, even in non-permissive daylengths. When this happens, it marks the end of the AVP and the start of the reproductive phase (RP) during which plants are committed to flower^1^,^2^. The length of the JVP and AVP can be established by experiments in which plants are transferred between inductive and non-inductive photoperiods or the reciprocal treatments at regular intervals following germination and recording flowering times of individual plants^3^,^4^,^5^,^6^,^7^. The times at which the transitions between photoperiod insensitive phases and the sensitive phase occur define the lengths of the JVP and AVP.

In plants, initiation of the reproductive phase is regulated by an elaborate network of floral signalling pathways, which include the photoperiodic, vernalization, autonomous, light-quality and ambient temperature pathways^8^,^9^. These ultimately regulate expression of the *FLOWERING LOCUS T* (*FT*) gene. In the photoperiodic pathway in *Arabidopsis FT* expression is rapidly induced by CONSTANS (CO) protein which is stabilised when high levels of *CO* expression coincide with light^10^. Flowering is promoted when FT protein is produced in permissive photoperiods and moves through the phloem to the apex where it forms a complex with FD and activates expression of the floral meristem identity genes^8^. The fact that plants are incapable of initiating flowering during juvenility even when environmental growth conditions are conducive suggests that inhibitory mechanisms may suppress induction of *FT* during juvenility and hence prevent premature flowering.

The B3 RAV (RELATED TO ABI3/VP1) sub-family is classified by the conserved WN/RSSQS motif found at amino acid position 245–250^11^. In *Arabidopsis*, 13 RAV genes have been classified and these are divided into 2 classes. Class I comprises six members that contain the APETALA2 (AP2) DNA binding domain in addition to the B3 domain^12^ and Class II contains 7 other less characterised genes. Four members of the RAV sub-family, RAV1, TEMPRANILLO 1 (TEM1), TEM2 and At3g25730, which all contain the C-terminal RLFGV motif, are proposed to act as transcription factors^13^. TEM1 and TEM2 have been shown to repress flowering acting redundantly to repress *FT* expression early in development through binding to two regions in the *FT* gene 5′ untranslated region^14^. Double mutant plants with reduced TEM1 and TEM2 activity flower earlier than the single *tem1* and *tem2* mutants, which flower earlier than WT plants^14^. Ectopic over-expression of both genes causes late flowering and *TEM1* over-expression almost completely suppresses *FT* expression^14^. In WT plants, *TEM1* mRNA is abundant in seedlings and declines before the floral transition when *FT* levels peak^14^. TEM1 and TEM2 are proposed to have a role in both the photoperiodic pathway, antagonising CO activity by competing for its binding site in the *FT* gene, and in the GA-dependent flowering pathway, repressing two GA_4_ biosynthetic genes, *GA3OX1* and *GA3OX2*, by binding a region in the first exon of both genes^14^,^15^. *TEM1* and *TEM2* are negatively regulated by APETALA1 (AP1) and GIGANTEA (GI)^16^,^17^.

As TEM has been proposed to inhibit flowering and to be expressed early in plant development, we hypothesised that TEM could perform a role in regulation of the JVP to AVP transition. The length of the JVP can vary greatly between species and between plants with short and long life cycles. In the model plant *Arabidopsis thaliana*, the JVP lasts for only a few days, which is very short compared to most species. To understand the wider significance of the regulation of juvenility in *Arabidopsis*, we also isolated *Arabidopsis TEM* orthologues from *Antirrhinum majus* L. (antirrhinum) and *Olea europaea* L. (olive). We use antirrhinum, which is a seed-raised crop with a LD photoperiod requirement and a well-characterised JVP of several weeks and olive, an important woody fruit crop, perennial tree species with a juvenile phase that lasts several years. We show that TEM from both species act as floral repressors and are expressed most highly during the juvenile phase of vegetative growth. In studies utilising *AmTEM* we show that *TEM* regulates juvenile phase length through a mechanism involving repression of both *FT* and *CO* genes.

## Results

### *Arabidopsis tem* mutants have a shorter JVP than wild type

**Figure 1:**
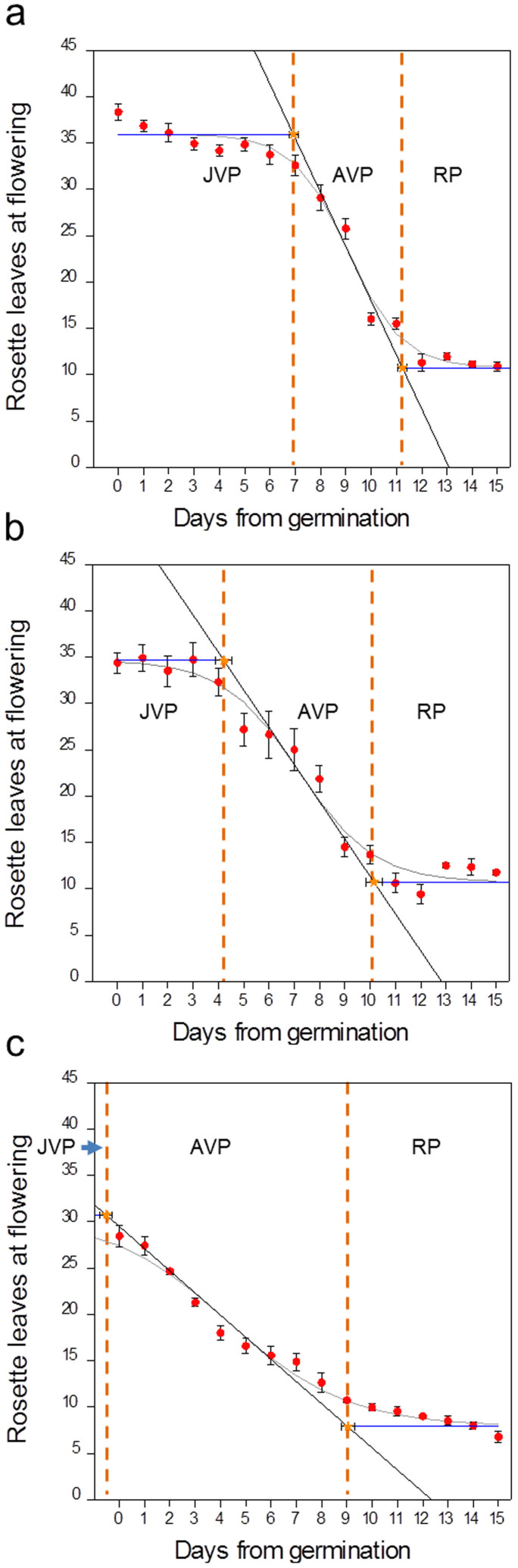
Different phases of photoperiod sensitivity in *Arabidopsis* Col-0, *tem1* and RNAi-*tem1*/2. The effect of transferring *Arabidopsis* at daily intervals (expressed as days from 50% germination) from LD to SD on flowering time in (a) Col-0, (b) *tem1* and (c) RNAi-*tem1*/2. JVP: juvenile phase, AVP: adult vegetative phase, RP: reproductive phase, SD: short day, LD: long day. The orange dotted lines delimit the three different phases. Vertical error bars denote the standard error of the mean of the number of leaves. Horizontal error bars denote the standard error of the mean of the estimated phase length. Logistic curve (grey curve), maximum slope (black line), lag time lines (blue horizontal lines). JVP: Col-0 vs. *tem1* p = 0.0104; Col-0 vs. RNAi-*tem1*/2 p = 0.0007; *tem1* vs. RNAi- *tem1*/2 p = 0.0029. AVP: Col-0 vs. *tem1* p = 0.042; Col-0 vs. RNAi- *tem1*/2 p = 0.0021; *tem1* vs. RNAi- *tem1*/2 p = 0.0072. Data were analysed by Tukey's multiple comparisons test after two-way ANOVA (p < 0.001).

Assessment of flowering times in *Arabidopsis* plants transferred from LDs to SDs at daily intervals from germination showed that the JVP lasted for 6.8 ± 0.2 d after germination in Col-0 wild-type plants, compared to 4.06 ± 0.35 d in the *tem1* single mutant and −0.5 ± 0.2 d, in the RNAi-*tem1*/2 double mutant (Fig. 1a–c). The lack of a measurable JVP in the double mutant indicates that TEM is required for *Arabidopsis* plants to express a juvenile phase and the intermediate length of the JVP in the single *tem1* mutant shows that levels of *TEM* influence the length of juvenility.

*TEM1* and *TEM2* messenger RNA (mRNA) levels were analysed in wild-type and *tem* mutant plants (Supplementary Fig. S1). Although some residual levels of *AtTEM1* were detected in the mutants, the levels were much lower than in the wild type and the levels of *TEM1* were not significantly different between the two mutants. Similar levels of *TEM2* were detected in *tem1* and wild-type plants but *TEM2* levels were considerably lower in RNAi-*tem1*/2 compared to *tem1* and wild-type plants. Overall, an inverse relationship between the amount of *TEM* expression and the length of the JVP was observed. *TEM* mRNA levels were shown to influence the length of the AVP; decreasing levels of *TEM* mRNA resulted in increased AVP lengths (Fig. 1a–c).

When grown under LD conditions, wild-type plants flowered later than *tem1* and RNAi-*tem1*/2 plants (Supplementary Fig. S1). Wild-type plants also flowered later than *tem1* and RNAi-*tem1*/2 plants in SDs (Supplementary Fig. S1). In LDs, the double mutant flowered even earlier than the single *tem1* but in SDs the flowering time of the single and double mutants was not significantly different. Thus although TEM acts as a floral repressor in both LD and SD conditions, it may not be through the same mechanism.

### Reciprocal changes in the expression of *TEM* and *FT* correlate with JVP length in *Arabidopsis*

**Figure 2:**
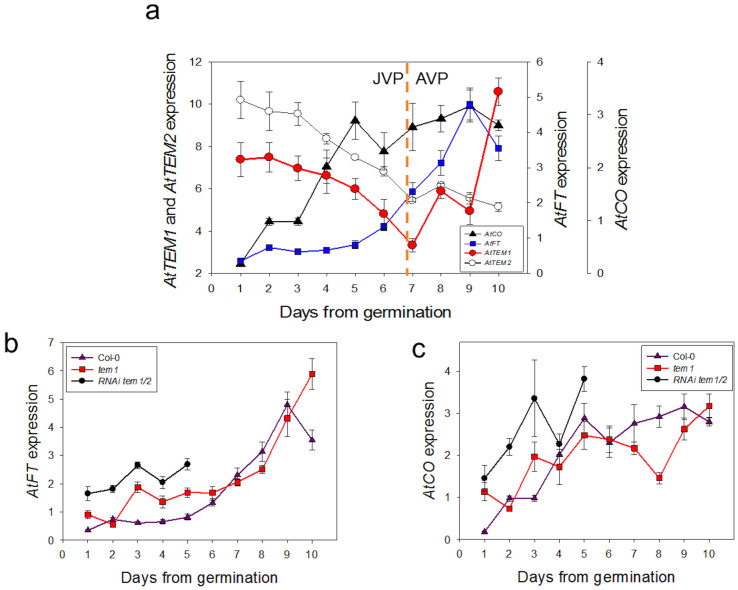
Relationship between *AtFT*, *AtCO*, *AtTEM1* and *AtTEM2* levels and juvenile phase length in Col-0, *tem1* and RNAi-*tem1*/2. (a) Real-time PCR analysis of developmental expression of *AtFT*, *AtCO*, *AtTEM1* and *AtTEM2* in aerial parts of Col-0 plants grown under LD harvested at ZT15. *AtFT*, *AtCO* *AtTEM1* and *AtTEM2* were normalised to *ACTIN2* and *β-TUBULIN* at each timepoint. (b) Real-time PCR analysis of developmental expression of *AtFT* in aerial parts of Col-0, *tem1* and RNAi-*tem1/2* plants grown under LD harvested at ZT15. *AtFT* was normalised to *ACTIN2* and *β-TUBULIN* at each timepoint. (c) Real-time PCR analysis of developmental expression of *AtCO* in aerial parts of Col-0, *tem1* and RNAi-*tem1*/2 plants grown under LD harvested at ZT15. *AtCO* was normalised to *ACTIN2* and *β-TUBULIN* at each timepoint. The error bars represent the standard error of the normalized relative quantities.

We investigated the relationship between levels of *TEM* and *FT* by examining gene expression changes occurring around the time of the JVP to AVP transition in wild type and *tem* mutants of *Arabidopsis*. Induction of *FT* expression in wild-type plants grown under LDs occurred just prior to the end of the JVP (Fig. 2a) and levels of *TEM1* and *TEM2* mRNA declined rapidly at the time when *FT* mRNA levels were shown to increase (Fig. 2a). *FT* was induced at an earlier stage in the *tem1* mutant and expressed even earlier and at a higher level in the double mutant (Fig. 2b). *TEM1* activity was observed to increase during the AVP in wild type, which may indicate that it has additional functions in older plants.

*CO* expression was used as a measure of activity of the photoperiodic flowering pathway to determine whether inactivity of the pathway could be the cause of reduced levels of *FT* expression during juvenility (Fig. 2a). *CO* mRNA levels started to rise within 2 days of germination and reached significant levels prior to the end of the JVP, and before the induction of *FT* expression. This suggests that the photoperiod pathway is active during the JVP and that activation of *FT* by *CO* during juvenility is prevented by the repression of *FT* by *TEM*. Comparison of *CO* mRNA levels in wild-type and RNAi-*tem1/2* plants showed that *CO* mRNA levels are themselves partially suppressed by TEM (Fig. 2c). These findings indicate that regulation of the photoperiodic flowering pathway during juvenility occurs by the repression of both *FT* and *CO* by TEM. The mechanism by which TEM represses CO is not known but analysis of the 5′ UTR region of *AtCO* showed the presence of CCACA and CATCTG sequences that could be considered variants of the motifs recognised and bound by AtTEM through its AP2 and B3 domains as described by Kagaya, *et al.*^18^ (Supplementary Fig. S2), raising the possibility that TEM might directly regulate *CO* expression.

### Reciprocal changes in the expression of *TEM* and *FT* correlate with JVP length in antirrhinum

**Figure 3:**
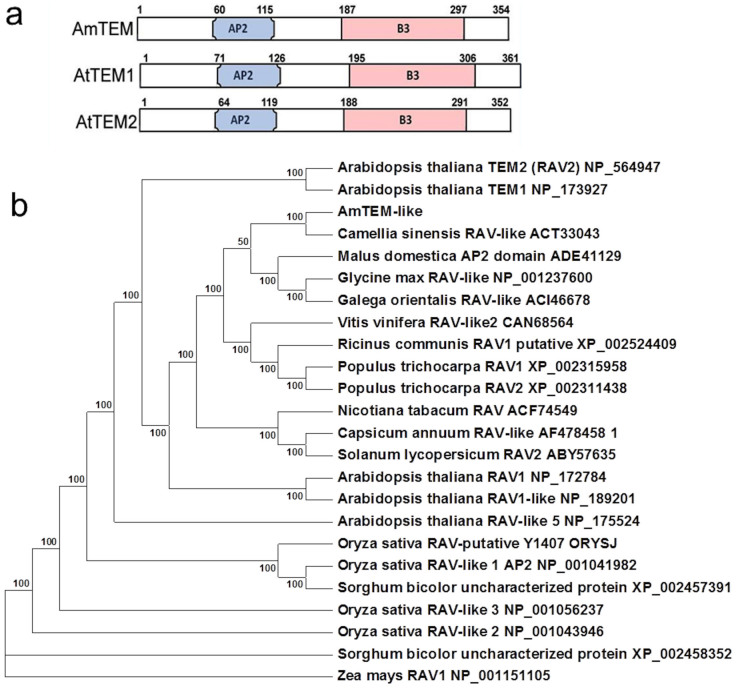
Relationship of RAV sub-family class I members with AmTEM. (a) Comparison of protein domain structure in AmTEM, AtTEM1 and AtTEM2. (b) Phylogenetic analysis of the deduced amino acid sequence of AmTEM and other RAV sub-family class I member homologs. The evolutionary relationship was inferred using the Maximum Parsimony method. The percentage of parsimonious trees in which the associated taxa clustered together are shown next to the branches. Accession numbers are given next to the species name.

To determine whether TEM may have a wider role as a regulator of juvenility beyond *Arabidopsis* we investigated its role in antirrhinum. A full-length *AmTEM* cDNA was obtained, consisting of a coding sequence of 1065 bp, predicted to encode 354 amino acids. The protein contained the AP2 and B3 domains that characterise the RAV class I protein family (Fig. 3a). Phylogenetic analysis using the deduced amino acid sequence showed that AmTEM is homologous to RAV-like sub-family class I DNA binding proteins from other organisms (Fig. 3b). Alignment to the *Arabidopsis* TEM proteins revealed sequence homology covering the length of the coding sequence with overall sequence identities of 68.7% and 68.2% with AtTEM1 and AtTEM2, respectively (Supplementary Fig. S3).

**Figure 4:**
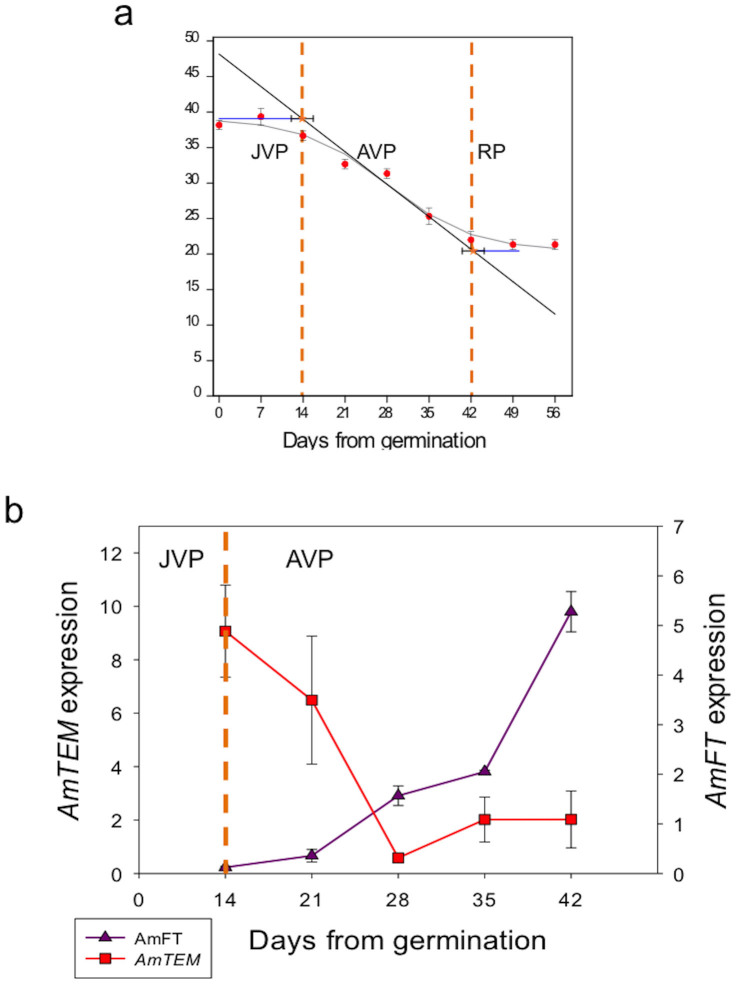
Different phases of photoperiod sensitivity in antirrhinum (Bells F1) and analysis of *AmTEM* and *AmFT* expression. (a) Different phases of photoperiod sensitivity in antirrhinum. The effect of transferring antirrhinum at weekly intervals (expressed as days from 50% germination) from LD to SD on flowering time. JVP: juvenile phase, AVP: adult vegetative phase, RP: reproductive phase, SD: short day, LD: long day. The orange dotted lines delimit the three different phases. Vertical error bars denote the standard error of the mean of the number of leaves. Horizontal error bars denote the standard error of the mean of the estimated phase length. Logistic curve (grey curve), maximum slope (black line), lag time lines (blue horizontal lines). (b) Real-time PCR analysis of developmental expression of *AmTEM* and *AmFT* in the youngest pair of fully expanded leaves in antirrhinum plants grown under LD harvested at ZT15. *AmTEM* and *AmFT* relative expression levels were normalised to *ACTIN* and *ELONGATION FACTOR 1 α ELF-alpha* at each timepoint. The error bars on the bars represent the standard errors of the normalized relative quantities.

Weekly transfers from LD to SD over a period of 8 weeks were carried out with antirrhinum plants and the number of leaves at flowering was used to calculate the length of the JVP and AVP. In these experiments, the JVP in antirrhinum was calculated to have ended 13.9 ± 1.8 d after germination (Fig. 4a). *AmFT* and *AmTEM* mRNA levels were measured in the youngest pair of expanded leaves, which would be the main source of assimilates for the apex, in plants grown under continuous LD at the time of transfer to SD. There was a clear reciprocal relationship between *AmFT* and *AmTEM* expression levels around the time of the JVP to AVP transition (Fig. 4b).

We also measured the levels of *AmFT* and *AmTEM* mRNA in all of the leaves at the time of transfer (Supplementary Fig. S4). *AmFT* expression was low during juvenility and progressively increased in all true leaves following the transition to an adult phase of growth. In contrast, *AmTEM* mRNA levels were high in the first three pairs of leaves after 14 and 21 days and then much lower in all leaves at later harvests indicating that TEM expression is a function of plant age rather than the age of individual leaves.

It has been proposed that in *Arabidopsis* the B3 and the AP2 domains in TEM are both necessary for its inhibition of *FT*. TEM is thought to bind to the 5′ UTR of *FT*, thus competing with CO for its binding site^14^,^18^. Investigation of the 5′ UTR region of *AmFT* showed that it harbours CAACA and GTCCTT regions that could be targeted for binding by AmTEM (Supplementary Fig. S5). Furthermore, a putative CO binding site is also present in the 5′ UTR region of *AmFT*, which lies between the B3 and AP2 putative binding sequences. Thus in antirrhinum a similar competing mechanism could exist for regulation of *AmFT* by AmCO and AmTEM.

### *AmTEM* functionally complements *AtTEM1* in the *tem1* mutant

**Figure 5:**
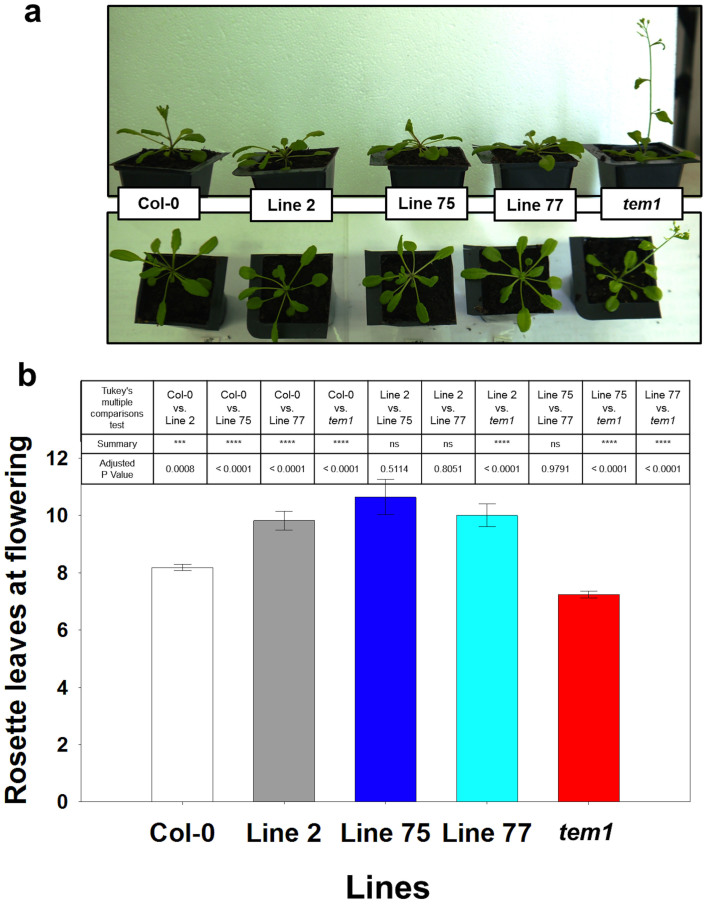
Phenotype of T_3_ generation *35S::AmTEM/tem1* line 75, line 2 and line 77 plants, the non-transformed *tem1* mutant and Col-0 grown under LD conditions. (a) Transgenic lines at 26 days from germination compared with the *tem1* mutant and Col-0. (b) Number of leaves at flowering time of the transgenic lines, *tem1* mutant and Col-0 plants. Data were analysed by Tukey's multiple comparisons test after two-way ANOVA (p < 0.001) (*** and **** = extremely significant; n.s = not significant). For Col-0, n = 68; for line 2, n = 11; for line 75, n = 11; for line 75, n = 14; for tem1 mutant, n = 68. Error bars represent the standard error of the mean.

*AmTEM* was ectopically expressed in the *tem1* mutant under the control of the *CaMV* 35S promoter. A total of 35 independent T_1 _*35S::AmTEM/tem1* transgenic lines were generated that all flowered later than *tem1* mutant plants when analysed under SD conditions. The majority of the lines also flowered later than Col-0 wild-type plants (Supplementary Fig. S6). *tem1*, wild-type and *35S::AmTEM/tem1* plants flowered at 32.4 ± 0.9, 37.9 ± 1.2 and 54.6 ± 1.8 rosette leaves, respectively. *35S::AmTEM/tem1* lines, lines 2, 75 and 77, were selfed and grown through to homozygosity in the T_3_ generation for subsequent analyses. Activity of the transgene was confirmed by detection of *AmTEM* mRNA in T_3 _plants representing each line (Supplementary Fig. S7). *AmTEM* expression levels were significantly higher in line 75 than line 77, with line 75 levels also being higher than in line 2. Line 77 *AmTEM* levels were lower than those in line 2. Late flowering phenotypes were maintained in the T_3_ generation and persisted in plants grown under LD conditions (Fig. 5a, b). The *35S::AmTEM/tem1* lines 2, 75 and 77 all flowered significantly later than *tem1* plants, however there was no significant difference between the flowering time of the three different lines. This indicated that the levels of *AmTEM* were saturating with respect to the effect on flowering time in all of the transgenic lines. Control plants engineered to over-express the *Arabidopsis TEM1* gene similarly exhibited late flowering (Supplementary Fig. S8).

### Over-expression of *AmTEM* prolongs juvenility and represses *FT* and *CO*

**Figure 6:**
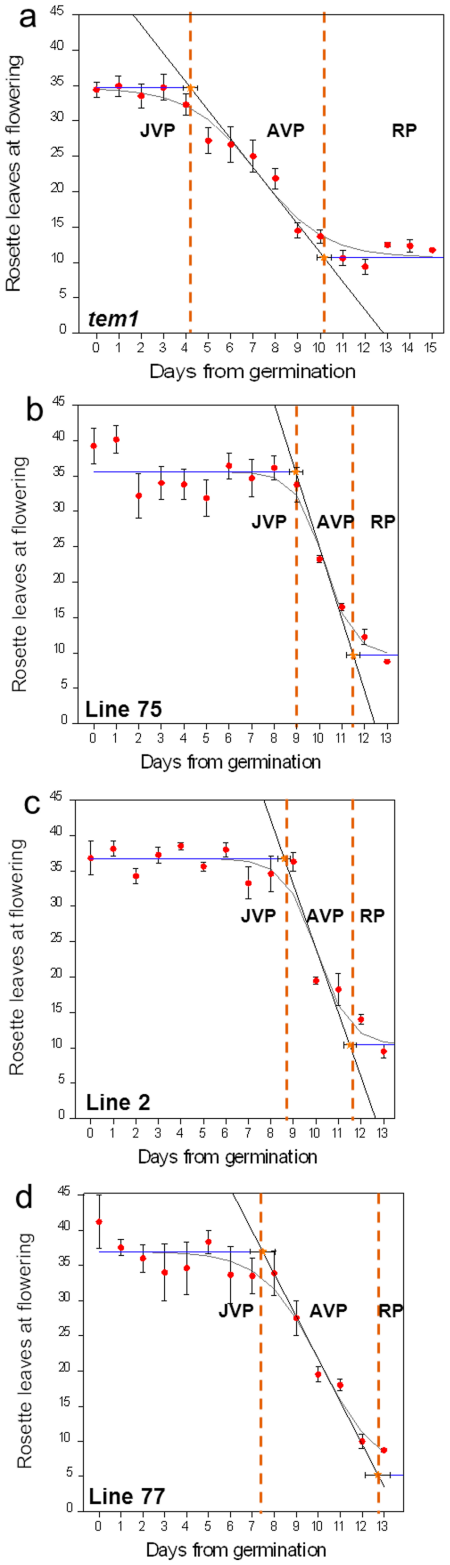
Different phases of photoperiod sensitivity in (a) *tem1*, (b) *35S::AmTEM/tem1* line 75, (c) *35S::AmTEM/tem1* line 2 and (d) *35S::AmTEM/tem1* line 77. The effect of transferring *Arabidopsis* at daily intervals (expressed as days from 50% germination) from LD to SD on flowering time. JVP: juvenile phase, AVP: adult vegetative phase, RP: reproductive phase, SD: short day, LD: long day. Vertical error bars denote the standard error of the mean of the number of leaves. Horizontal error bars denote the standard error of the mean of the estimated phase length. Logistic curve (grey curve), maximum slope (black line), lag time (blue lines). The orange dotted lines delimit the three different phases. JVP: *tem1* vs. line 75 p = 0.0032; *tem1* vs. line 2 p = 0.0043; *tem1* vs. line 77 p = 0.0128; line 75 vs. line 2 p = 0.9048; line 75 vs. line 77 p = 0.1574; line 2 vs. line 77 p = 0.298. AVP: *tem1* vs. line 75 p = 0.011; *tem1* vs. line 2 p = 0.0165; *tem1* vs. line 77 p = 0.606; line 75 vs. line 2 p = 0.9048; line 75 vs. line 77 p = 0.025; line 2 vs. line 77 p = 0.0405. Data were analysed by Tukey's multiple comparisons test after two-way ANOVA (p < 0.001).

As lower *TEM* expression levels lead to shortening of the JVP we hypothesised that over-expression of *TEM* should lead to an increase in JVP length and that this should also affect patterns of *CO* and *FT* expression. JVP lengths of all three *35S::AmTEM/tem1* lines were longer than the JVP measured in *tem1* plants (Fig. 6a–d). Juvenility ended 4.06 d ± 0.35 d after germination in *tem1* plants (Fig. 6a), whilst in *35S::AmTEM/tem1*lines 75, 2 and 77 it ended 8.9 ± 0.26 d, 8.6 ± 0.26 d and 7.4 ± 0.33 d after germination respectively (Fig. 6b–d). The JVP lengths of the three transgenic lines were not significantly different, however, all were significantly longer than the JVP of *tem1*. In addition to having a shorter JVP, *tem* mutants exhibited a longer AVP. We therefore looked at the length of the AVP in the overexpressing lines. Compared to the *tem1* control, the AVPs in *35S::AmTEM/tem1* line 75 and *35S::AmTEM/tem1* line 2 were shorter but not significantly different from each other. The length of the AVP in *35S::AmTEM/tem1* line 77, the line with the lowest *AmTEM* levels, was similar to that in *tem1* plants, and longer than the more highly expressing lines. Thus, the expression level of *AmTEM* was sufficient in all lines to saturate effects on the JVP, but insufficient in line 77 to shorten the AVP.

**Figure 7:**
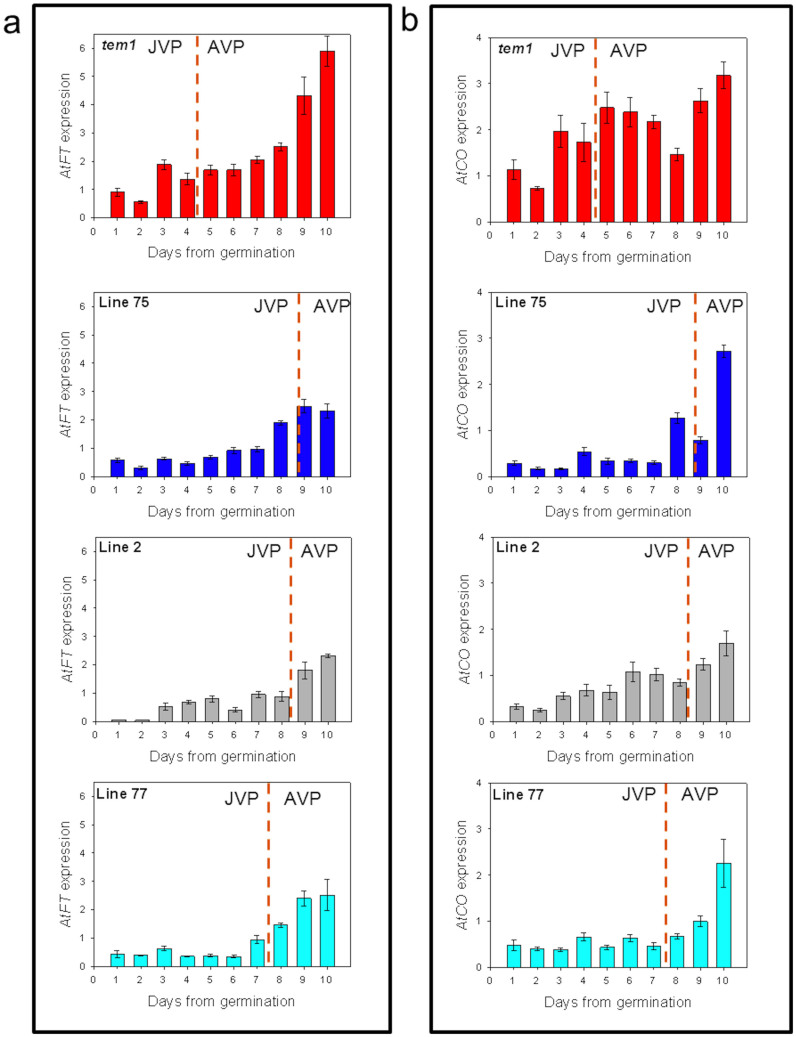
Real-time PCR analysis of developmental expression of (a) *AtFT* and (b) *AtCO* in *tem1* and T_3_ generation *35S::AmTEM/tem1* line 75, line 2 and line 77 plants. Plants were grown under LD conditions and aerial parts harvested at ZT15. *AtFT* and *AtCO* were normalised to *ACTIN2* and *β-TUBULIN* at each timepoint. The error bars on the bars represent the standard error of the normalized relative quantities. JVP: juvenile phase, AVP: adult vegetative phase. The orange dotted lines delimit the two different phases.

Overexpression of *AmTEM* inhibited *AtFT* and *AtCO* mRNA expression in all three lines during early development, compared to the *tem1* controls, and was accompanied by an extension in the length of the JVP (Fig. 7 a, b). In all the transgenic lines over-expressing *AmTEM* and in *tem1*, the increase in *AtFT* mRNA levels occurred around the end of the JVP. A similar pattern of expression of *AtCO* to *AtFT* mRNA was seen in the *35S::AmTEM/tem1* lines, with *AtCO* mRNA increasing around the end of the JVP. However, in the *tem1* plants, *AtCO* mRNA levels were high during juvenility and after it ended while being suppressed in the transgenic lines. This is consistent with the results from the *tem1* mutants that suggested *TEM* regulates *CO* in addition to *FT*.

### Olive *TEM* (*OeTEM*) is expressed predominantly during juvenility and is a floral repressor

**Figure 8:**
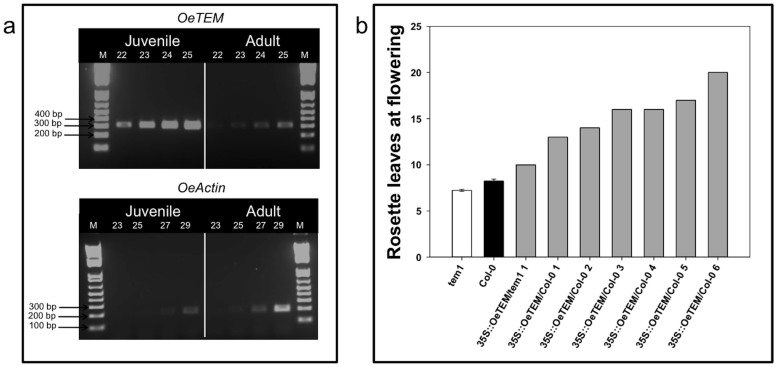
(a) Semi-quantitative analysis of *OeTEM* expression. Number of cycles used to amplify *OeTEM* and *OeACTIN* fragments are indicated. M = 1 kb Plus DNA ladder, Juvenile = juvenile olive leaf, Adult = adult olive leaf. (b) Flowering time of Col-0, *tem1* and T_1_ generation *35S::OeTEM* lines grown under LD conditions. Flowering time assessed by the number of rosette leaves when the bolt was 1 cm in length. Independent T_1_ transgenic lines (grey bars), Col-0 (black bar, n = 68) and *tem1* mutant (white bar, n = 68). Error bars represent the standard error of the mean.

To determine whether *TEM* is more wide-spread in the plant kingdom, we isolated and partially characterised a full-length cDNA representing an olive *TEM* ortholog (*OeTEM*) that contained AP2 and B3 domains and shared 64.6% and 66.1% identity at the amino acid level to *Arabidopsis* TEM1 and TEM2 proteins respectively (Supplementary Fig. S9). *OeTEM* was expressed more highly in leaves from juvenile olive plants compared to adult (Fig. 8a), which supported a role for it functioning during juvenility.

Flowering times of *35S::OeTEM* T_1_ transgenic lines, engineered to over-express *OeTEM* in *tem1* and Col-0 wild-type backgrounds were delayed. When grown under LDs, wild-type and *tem1* plants initiated flowering at 8.2 ± 0.11 and 7.2 ± 0.12 rosette leaves, respectively, whereas all *35S::OeTEM* lines flowered later at an average of 15.1 rosette leaves (Fig. 8b). Therefore, OeTEM functionally complemented the *tem1* mutant, and acts as a repressor of flowering in Arabidopsis.

## Discussion

Using transfer experiments from LD to SD we found that the JVP in the *Arabidopsis* ecotype Col-0 lasts for about 6–7 days. During the JVP, *FT* mRNA levels are low and they increase around the transition to the AVP, which is consistent with the JVP being the result of an inability to express *FT*. *CO* mRNA levels increase several days earlier than *FT*, which indicates that the photoperiod pathway for controlling flowering time is functional during the early stages of development and that an additional factor represses *FT* during the JVP.

The properties we predict for such a repressor of *FT* are that it would be a floral repressor, it would exhibit a complementary pattern of expression to *FT*, at least during the JVP, and that mutants impaired in repressor activity would result in a shorter JVP. All three requirements are met by TEM. *TEM* expression was high immediately after germination and fell to a low level by the end of the JVP. These observations are in line with findings of Castillejo and Pelaz^14^ and Osnato *et al.*^15^ who also found complementary changes in *AtTEM* and *AtFT* during early development in *Arabidopsis*, although they did not relate this to the juvenile/adult phase change. In our experiments, we found that the single *tem1* mutant had a shorter JVP than the Col-0 control and in the RNAi-*tem1*/2 double mutant there was no measureable JVP. Thus TEM is essential for *Arabidopsis* to show a JVP and the level of *TEM* expression affects the length of the JVP.

Antirrhinum is a seed raised flower crop that is a facultative LDP with a well-defined juvenile phase for flowering. We studied whether TEM was also involved in the regulation of the JVP in antirrhinum by isolating a *TEM* orthologue (*AmTEM*) and testing its properties. Sequence analysis identified *AmTEM* as a member of the B3 super-family, family RAV, class I. It contains a B3 domain, which includes the WN/RSSQS motif, which is characteristic of the RAV family, and the AP2 domain that defines Class I genes as proposed by Romanel *et al.*^12^. Phylogenetic analysis showed that *AmTEM* is closely related to other RAV-like DNA binding proteins clustering with AtTEM1 and AtTEM2 and sharing close homology to the related AtRAV1. However, while *AtRAV1* plays a role in leaf senescence^19^, no signs of premature senescence were visible in any of the transformed plants that were engineered to over-express *AmTEM*, *OeTEM* or *AtTEM1*. In addition, transgenic plants with reduced or increased RAV1 expression were reported as having no significant difference in leaf number in LD^20^, in contrast to the TEM mutants or overexpressors, which show reduced or increased leaf number respectively (e.g. Fig. 5a, Supplementary Fig. S9). We conclude that the *AmTEM* and *OeTEM* genes isolated in this study are likely to be TEM rather than RAV genes.

In antirrhinum, the pattern of expression of *AmTEM* and *AmFT* is consistent with TEM having a role in regulating the JVP by repressing *FT*. A reciprocal relationship between *AmTEM* and *AmFT* was observed, with levels of *AmTEM* being high during early development and decreasing at the end of juvenility, after which *AmFT* levels increase. The continuing reciprocal changes in TEM and FT after the end of the JVP are consistent with an increasing sensitivity to LDs as the plants age^21^. We also found that the changes in *TEM* mRNA levels were a function of plant age, rather than leaf age, which again is consistent with TEM having a specific role in establishing the JVP.

*AmTEM* complemented both the floral repressor and JVP regulator functions of the *Arabidopsis* *TEM1* gene when overexpressed in the *tem1* mutant. Plants over-expressing *AmTEM* were late flowering in both LD and SD when compared to the *Arabidopsis tem1* single mutant and WT. Overexpressing *AmTEM* in *Arabidopsis* resulted in extension of the length of the JVP by up to 5 days. However, the combined JVP and AVP was less affected, being extended by only about 3 days, suggesting that TEM had its biggest effect early in development. A gradation of responses to TEM, including the JVP, was shown in the transgenics with different levels of expression. Taken together with the observation that the single TEM mutant of *Arabidopsis* had a JVP intermediate between the double mutant and the WT, we conclude that the length of the JVP is linked to the level of TEM expression. However, even in the highest over-expressors, there was still a measureable JVP and AVP. This suggests that an additional level of regulation of TEM, possibly post-translational, is involved in its control of the JVP. The expression of *FT* and *CO* was also suppressed in the transgenics, confirming that TEM represses the expression of *CO*, as well as its target *FT*.

Juvenility has been mostly studied in herbaceous species where it usually lasts for a relatively short time. However, JVP length can be dramatically extended in woody species, such as olive, varying from 1 to 20 or more years^22^,^23^,^24^,^25^,^26^,^27^,^28^,^29^. We are interested in the extent to which mechanisms establishing a JVP in herbaceous plants are conserved in woody species. A full-length cDNA representing a *TEM* orthologue was isolated and characterised from olive. *OeTEM* was shown to contain AP2 and B3-like domains characteristic of the RAV family. Phylogenetic analysis showed that both AmTEM and OeTEM are closely related to other RAV-like DNA-binding proteins clustering with AtTEM1 and AtTEM2 and sharing close homology to the related AtRAV1. In olive, initial results show that *OeTEM* expression levels are higher during juvenility than when plants are adult. *Arabidopsis* plants over-expressing *OeTEM* were delayed in flowering, confirming that *OeTEM* is a floral repressor, with similar functional properties to TEM from both *Arabidopsis* and antirrhinum.

Castillejo and Pelaz (2008) proposed that *AtTEM1* and *AtTEM2* genes show functional redundancy in regulating *FT* expression and this is supported by the results presented in this paper. *AtTEM1* and *AtTEM2* have an additive effect of regulating the JVP and in the absence of *AtTEM1,* *AtTEM2* cannot fully compensate for it in maintaining juvenility. However, *AtTEM1* and *AtTEM2* have been shown to function independently in a different response. *TEM2* was proposed be a requirement for blocking RNA silencing by two distinct viral proteins and it was shown that the *tem2* mutant could not be functionally complemented by *AtTEM1* for this response^30^.

AtTEM inhibits flowering in both LD and SDs. While the inhibition in LDs may be linked to an extended JVP, this would not be the case for SDs. Osnato et al. (2012) have shown that *TEM* genes directly repress the expression of the GA(4) biosynthetic genes *GA 3-oxidase1* and *2* (*GA3OX1* and *GA3OX2*), by binding to a regulatory region in the first exon, and thus inhibiting GA-dependent flowering in SDs. In this paper we present evidence that TEM not only antagonises CO in regulating *FT* as previously proposed, but also represses its expression.

As mentioned previously, plants overexpressing TEM still exhibit a JVP and AVP, indicating there may be additional, higher levels of control of phase change. Molecules involved in vegetative phase change in a range of species have been identified, including microRNA156 (miR156) and miR172. miR156 is expressed at high levels in young tissues and decreases significantly during development^31^,^32^,^33^,^34^. During the juvenile phase, miRNA156 acts to repress members of the SBP/SPL transcription factor family, which in turn target transcription factors including AP1, AGL42, LFY, FUL and SOC1^35^. miR172, which promotes competence to flower, is repressed by SPL genes that are direct targets of miR156 and consequently shows a reciprocal pattern of increasing expression with age^36^. Overlaid on this general pattern of age-related phase change, TEM can be considered as a floral repressor that acts on multiple points in the photoperiod and GA flowering pathways. TEM is required to establish and control the length of a JVP in *Arabidopsis* and TEM orthologues from antirrhinum and olive retain this function. Taken together with patterns of temporal expression in these species, we propose that TEM may have a more general role in regulating juvenility in a range of herbaceous and woody species.

## Methods

### Plant Material

F_1_ seeds of *Antirrhinum majus* L. (antirrhinum), cv. Bells Red, were obtained from Goldsmith Seeds, Inc. (Syngenta Flowers-Gilroy, CA). Seeds of the *Arabidopsis thaliana* Columbia (Col-0) ecotype and *tem1* mutant in the Col-0 background (SALK_097513) were obtained from the Nottingham *Arabidopsis* Stock Centre (NASC). Seeds of *Arabidopsis* RNAi-*tem1/2* double mutant (line 94.9, T5–T6 generations) in the Col-0 background were kindly donated by Dr Soraya Pelaz Herrero (Centre de Recerca Agrigenòmica, SPAIN).

*Olea europaea* L. (olive) leaf samples were collected from trees of cv Leccino grown in the agricultural farm of Tuscia University (Italy) on the 25th of June 2010 at 20:00 (sun rise was at 5:34, sun set was at 20:52), frozen in liquid nitrogen and stored at −80°C until their utilization. Total RNA was isolated using the RNeasy Plant Mini Kit (QiagenInc, Cat. No. 74903, UK). The juvenile leaves were sampled from a seedling with juvenile characteristics that had never flowered. Adult leaves were sampled from an adult plant.

Antirrhinum and *Arabidopsis* plants were grown in SANYO MLR-351H growth chambers set at 22°C, 70 ± 2% relative humidity (RH) and short days (SD) (8 h photoperiod). When 50% of seedlings had emerged they were placed under the appropriate light conditions at 22°C. Lighting in SD conditions consisted of 8 h of fluorescent light (DLI = 2.94 mol m^−2^ d^−1^). LD conditions were achieved using a combination of 8 h of fluorescent light (DLI = 2.79 mol m^−2^ d^−1^) and an extension of 8 h of tungsten light (Philips 32W, NL) (DLI = 0.29 mol m^−2^ d^−1^) totalling 3.08 mol m^−2^ d^−1^. Light quality and quantity were measured with an EPP 2000 Fiber Optic Spectrometer (StellarNet Inc. USA).

### Antirrhinum and *Arabidopsis* transfer experiments

In antirrhinum transfer experiments, plants were moved from LD to SD every 7 days for 8 weeks. Transfers started when 50% of the seedlings germinated (T_0_) and transferred plants remained under SD conditions until flowering. In the *Arabidopsis* transfer experiments plants were moved from LD to SD every day, from 50% germination (T_0_). Flowering time in antirrhinum was measured as the number of true leaves present under the inflorescence. Flowering in *Arabidopsis* was measured as the number of rosette leaves when the bolt was at 1 cm length. A further 10 plants, during each transfer experiment, were grown in constant SD and LD conditions as controls. Analysis of the flowering time data from the transfer experiments to determine the different phases of photoperiod sensitivity was performed with GenStat (thirteenth edition)^37^.

### Gene isolation

Leaf material was harvested from antirrhinum when plants were 12 days (juvenile) and 24 days (adult) old and pooled. Total RNA was extracted using Trizol® reagent (Invitrogen Ltd., Cat. No. 15596-026), following the manufacturers' guidelines. Samples were DNase treated using TURBO DNA-free™ (AmbionInc, Cat. No. AM1907) and first-strand cDNA synthesised using Superscript™ II Reverse Transcriptase (Invitrogen Ltd., Cat. No. 18064-14) following the manufacturers' guidelines.

A partial sequence of antirrhinum *TEMPRANILLO* (*AmTEM*) was isolated by PCR using cDNA and degenerate primers. To aid the design of degenerate primers the sequences of *Arabidopsis* TEM1, TEM2 and RAVs were obtained from the TAIR database (http://www.arabidopsis.org/) and the Basic Local Alignment Search Tool (BLAST) from the National Center for Biotechnology Information (NCBI) GenBank database (http://www.ncbi.nlm.nih.gov/) was used to identify other sequences with high sequence homologies. Amino acid sequences used for the alignment were RAVs and RAV-like from different species selected for high homology to the *Arabidopsis* RAV family genes. Primers were based on aligned amino acid sequences found in AtTEM1 and AtTEM2 in conserved regions and the Codon Usage Database (http://www.kazusa.or.jp/codon/) for antirrhinum used to determine nucleotides to incorporate into degenerate primers (Supplementary Fig. S10). Primers were designed to anneal to different regions of the gene covering almost all the entire TEM1/2 sequence (Supplementary Fig. S11). PCR products of the expected lengths were purified, using QIAquick® gel Purification Kit (Qiagen, Australia) and ligated into the pGEM-T Easy vector. Ligated vector products (2 μl) were added to 20 μl of electrocompetent EC100 E. coli cells and electroporated. Isolated plasmid DNA was sequenced using M13 primers (Supplementary Tab. S1). Contigs were obtained from sequenced fragments, using the Seqman package of DNAStar (DNAStar Inc.). New primers, CI-AmF and CI-AmR, specific for the 5′- and 3′- end of the contig and representing the middle portion of the gene, were designed and used in PCR to isolate the entire contig as a single fragment (Supplementary Tab. S1).

An EST sequence representing a full-length cDNA of the antirrhinum *FT* ortholog (*AmFT*) (AJ803471) was sourced from the antirrhinum sequence database DragonDB (http://www.antirrhinum.net/). AmFT shared 78.3% identity at the amino acid level to FT and was shown to functionally complement the *Arabidopsis ft-1* late-flowering mutant to restore early flowering (Supplementary Fig. S12).

A partial sequence (singleton f7khmq104im4eu) of a putative olive *TEM* (*OeTEM*) was identified by performing a BLAST search of an olive floral EST library obtained by 454-transcription sequencing using the *Arabidopsis TEM1* and *TEM2* AP2 domains.

RACE PCR was used to obtain 5′- and 3′ end sequence information of the *AmTEM* and *OeTEM*, sequences using a Gene Racer kit following the manufacturers' guidelines (Invitrogen Ltd., Cat No. L1502-02). Full-length cDNAs representing *AmTEM* (JX997989), *OeTEM* (KC007944) and *AtTEM1* were obtained using AmTEM1F/AmTEM1072R, OeTEM1F/OeTEM1074R and AtTEM1-F/AtTEM1091-R primers, respectively (Supplementary Tab. S1).

### Real-time PCR analysis

Total RNA was extracted using Trizol® reagent (Invitrogen Ltd., Cat. No. 15596-026), following the manufacturers' guidelines. Leaf material was harvested at ZT15 from seven replicate plants to generate each antirrhinum sample and 10 replicate seedlings to generate each *Arabidopsis* sample. Samples were DNase treated using TURBO DNA-free™ (Ambion Inc, Cat. No. AM1907). First-strand cDNA was synthesised using Superscript™ II Reverse Transcriptase (Invitrogen Ltd., Cat. No. 18064-14) following the manufacturers' guidelines. Real-time PCR analysis was conducted using the CFX384 Touch™ Real-time PCR machine (Bio-Rad Laboratories Ltd., UK). Each reaction contained 5 μl Sso Advanced™ SYBR® Green Supermix (Bio-Rad Laboratories Ltd., UK), 0.5 μl of cDNA, appropriate primer concentration (Supplementary Tab. S1), in a total volume of 10 μl made up with SDW. Quantitative Real-time PCR was performed using three technical replicates for each sample.

*ACTIN* (HQ853640) and *ELONGATION FACTOR 1 α* (AJ805055) were used as reference genes for the expression analysis in antirrhinum and *ACTIN2* (BE038458) and *β-TUBULIN* (AY040074) were used as reference genes in *Arabidopsis* analysis (Supplementary Tab. S1). After PCR amplification, all products were sequenced to confirm their identity.

Analyses were conducted according to MIQE guidelines^38^. Normalised gene expression levels was determined by the geometric mean of the relative quantities for all reference targets using target and run specific amplification with qBase Plus software version 2.5 (http://www.biogazelle.com/qbaseplus) (Supplementary Tab. S2).

### Semi-quantitative PCR

First-strand cDNA was synthesised using Superscript™ II Reverse Transcriptase (Invitrogen Ltd., Cat. No. 18064-14) following the manufacturers' guidelines using 3 μg total RNA for each sample. cDNA samples used for the olive analysis represent a juvenile and an adult sample. The amplification consisted of an initial denaturation at 94°C for 2 min, denaturation at 94°C for 15 s, annealing for 30 s, and extension at 72°C for 30 s for a range of cycles, comprising between 15 and 40. Primers, cycle ranges and annealing temperatures used to detect each gene are shown in Supplementary Table S1.

### Amino acid sequence comparisons and phylogenetic analysis

The deduced amino acid sequences of AmTEM and OeTEM were aligned with 23 RAV sub-family class I members using Clustal W MegAlign package of DNAStar (DNAStar Inc.). Evolutionary relationships of RAV sub-family members were inferred using the Maximum Parsimony method. Bootstrap values were derived from 500 replicate runs.

### *Agrobacterium*-mediated transformation of *Arabidopsis* with *AmTEM*, *OeTEM* and *AtTEM1*

Cloning of *AmTEM*, *OeTEM* and *AtTEM1* sequences was achieved using the Invitrogen gateway technology, using pDONR207 (Invitrogen®) as the entry clone for each coding sequence. The genes were then cloned, using Gateway® LR Clonase® II (Invitrogen, USA), into a pB2GW7 binary vector (Invitrogen Ltd., USA) to produce the pBAmTEM, pBOeTEM and pBAtTEM1 vectors.

*Agrobacterium* harbouring pBAmTEM, pBAOeTEM or pBAtTEM1 vectors were used to transform 10 Col-0, and 10 *tem1 Arabidopsis* plants using the floral dip method^39^. T_0_ plants were grown in Sanyo MLR growth chambers and T_1_ seeds collected. *Arabidopsis* plants transformed with *AmTEM* gene were sown, stratified and grown under SD conditions, while transgenic plants transformed with *OeTEM* and *AtTEM1* were grown under LD conditions. Plants were sprayed every 2 weeks from emergence of the first true leaves, with the BASTA herbicide (Bayer Crop Science, Cat. No. 05936136), containing Glufosinate-ammonium at a concentration of 150 mg l^−1^. The resistant plants were allowed to flower. Flowering times were recorded. Genomic DNA from T_1_ plants was extracted from each plant to confirm the presence of the transgenes using the gene specific primers seqAmtem F/seqAmtem R, seqOetem F/seqOetem R and seq Attem1 F/seq Attem1 R to amplify *AmTEM*, *OeTEM* and *AtTEM1*, respectively (Supplementary Tab. S1). T_2_ seeds were collected from each T1 plant and sown and grown under constant LD conditions until flowering. For each line, one T2 plant was selected to generate T3 plants for further analysis.

## Author Contributions

T.S., R.M., B.T. and A.M. designed the experiments. T.S. and A.J. performed the experiments. T.S., B.T. and A.M. wrote the manuscript.

## Supplementary Material

Supplementary Information Supplementary Information
