# Supplementary material for: TEMPRANILLO is a regulator of juvenility in plants
Source: Sci Rep. 2014 Jan 15;4:3704. doi: 10.1038/srep03704 (PMC3892441; doi:10.1038/srep03704)
Supplement: Supplementary Information [file srep03704-s1.doc]

***TEMPRANILLO* is a regulator of juvenility in plants**

Tiziana Sgamma^1^, Alison Jackson^1^, Rosario Muleo^2^, Brian Thomas^1,*^and Andrea Massiah^1^

^1^School of Life Sciences, University of Warwick, Gibbet Hill Road, Coventry, West Midlands CV4 7AL, UK

^2^ Department of Agriculture, Forests, Nature and Energy, University of Tuscia, Via San Camillo de Lellis snc, Viterbo 01100, Italy

*For correspondence (e-mail Brian.Thomas@warwick.ac.uk, tel +44 24 7657 5050, fax +44(0)2476 522052).

**Supplementary Figure S1. a)** **Real-time quantitative PCR analysis of *AtTEM1* and *AtTEM2* in Col-0, *tem1* and RNAi-*tem1/2* plants grown under LD.** Aerial parts were harvested at ZT15. *AtTEM1* and *AtTEM2* relative expression levels were normalised to *ACTIN2* and *β-TUBULIN*. Expression was analysed in samples at daily intervals from germination over the first 5 days. The average of the first 5 days from germination is presented for each line. The error bars represent the standard error of the normalized relative quantities. **b)** **Flowering time of Col-0, *tem1* and RNAi-*tem1/2* plants.** Flowering time was assessed by number of rosette leaves when the bolt was 1 cm in length in Col-0 (LD n = 68; SD n =16), *tem1* (LD n = 68; SD n = 14) and RNAi-*tem1/2* (LD n = 68; SD n = 14) plants grown under LD and SD conditions. Error bars represent the standard error of the mean. Data were analysed by Tukey's multiple comparisons test after two-way ANOVA (p < 0.001) (**** = extremely significant; *** = extremely significant; ** = Very significant; n.s = not significant).

**Supplementary Figure S2.** ***AtCO* nucleotide sequence and AtTEM1 putative binding sites.** The start codon is indicated by red font. Putative TEM binding sites that are present in the 5’ UTR region are highlighted in yellow.


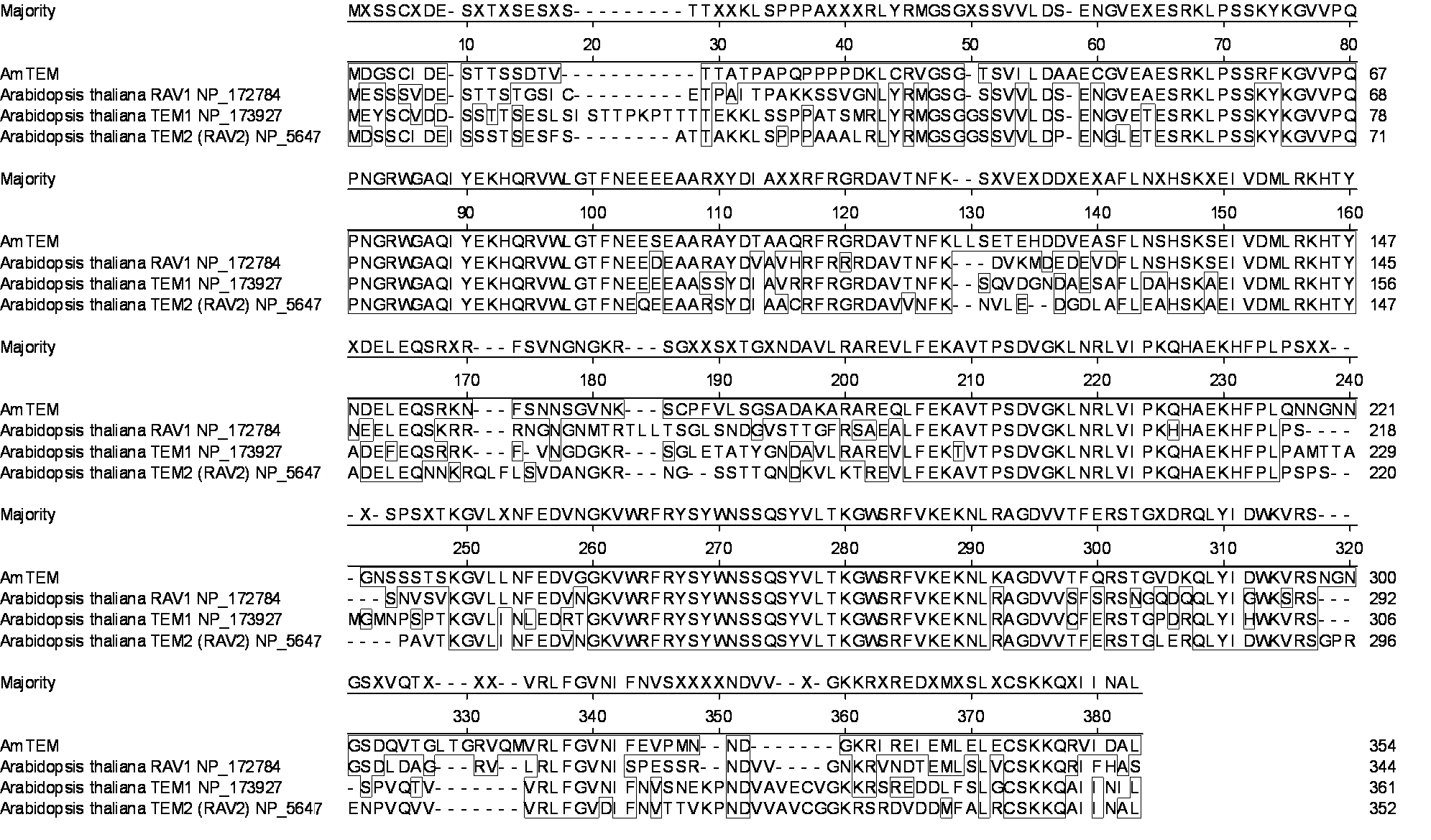


**Supplementary Figure S3. Amino acid sequence comparisons of AmTEM and related proteins in *Arabidopsis thaliana*.** Alignment of deduced amino acid sequences of AmTEM and AtRAV1, AtTEM1 and AtTEM2. Residues that are conserved with AmTEM are boxed.


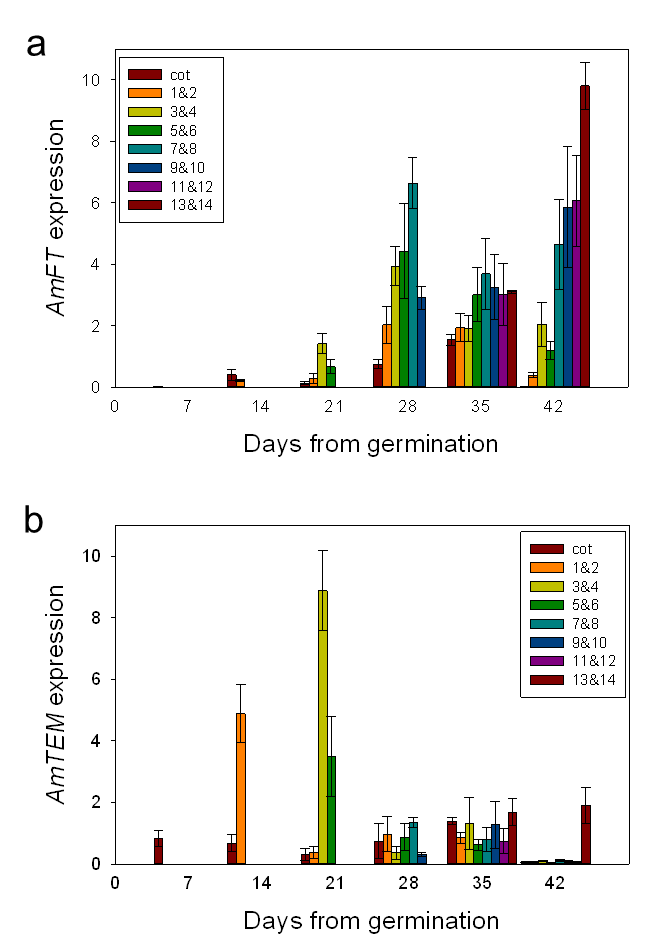


**Supplementary Figure S4.**  **Real-time PCR analysis of developmental expression of *AmTEM* and *AmFT* in all the leaves in *Antirrhinum* plants grown under LD harvested at ZT15.** a) Developmental expression of *AmFT* and b) *AmTEM* in leaf tissue from antirrhinum plants grown under LD harvested at ZT15. Real-time PCR analysis of the relative expression of both *AmFT* and *AmTEM* normalised to *ACTIN* and *ELONGATION FACTOR 1 α* at each timepoint. The error bars represent the standard errors of the normalized relative quantities.

> Antirrhinum majus AmFT (EM:AJ803471)

GAAACAACATAACTTGTCCTTCTATATAGTATTTTCATATAAAATTATAC

ATTTATATATCTTTCTTATCGTCATTAATTATAATTTAGACAAAAAAAAA

ATGCCTAGAGATAGGGATCCACTGGTGGTGGGAAGAGTGATAGGAGAAGT

ATTGGAGCCTTTCACGAGATCAATAGGGCTGAGAGTGATCTATAACAACA

GAGAAGTAAGCAATGGTTGTGATTTAAGGCCCTCTCAAGTTGTCAACCAA

CCTAGGGTTGAGATTGGAGGGGATGATCTCCGCACCTTCTACACTTTGGT

TATGGTGGACCCTGATGCTCCAAGTCCTAGTGACCCGAGTCTTAGGGAAT

ACTTACACTGGTTGGTGACTGATATCCCAGCAACCACCGGAACAAACTTC

GGTCAAGAGATTGTGTGTTATGAGAATCCACGGCCGTCGATGGGGATTCA

CCGCTTTGTTTTCACACTATTCCGCCAGTTGGGGCGGCAAACGGTGTACC

CTCCGGGTTGGCGCCAGAATTTCAACACGAGAGACTTTGCTGAGCTATAC

AACCTTGGCGCCCCAGTTGCTGCTGTCTACTTCAATTGCCAGAGGGAGAG

TGGTACCGGCGGGAGACGACGATAACGTCGAATTCGATCTCAATAATAGA

TCGATAAATAAAAATCATTTGATGGAATGTCAGTTTCGATTTTATCAATA

GTTGATCAAGTAGGAATCTTCATGCTTTG

**Supplementary Figure S5. *AmFT* nucleotide sequence and TEM putative binding sites.** The start codon is indicated by red font. Putative TEM binding sites that are present in the 5’ UTR region are highlighted in yellow. The putative binding site of CO and the CCAAT-box binding protein is highlighted in green.

**Supplementary Figure S6. Flowering time of *tem1*, Col-0 and T_1_ generation *35S::AmTEM****/****tem1*  lines grown under SD conditions.** Flowering assessed by number of rosette leaves when the bolt was 1 cm in length. T_1_ generation *35S::AmTEM/tem1*  lines (grey bars), *tem1* mutant (white bar, n = 14), Col-0 (black bar, n = 16). The hashed dark grey bars show the lines selected for further studies. Error bars represent the standard error of the mean.


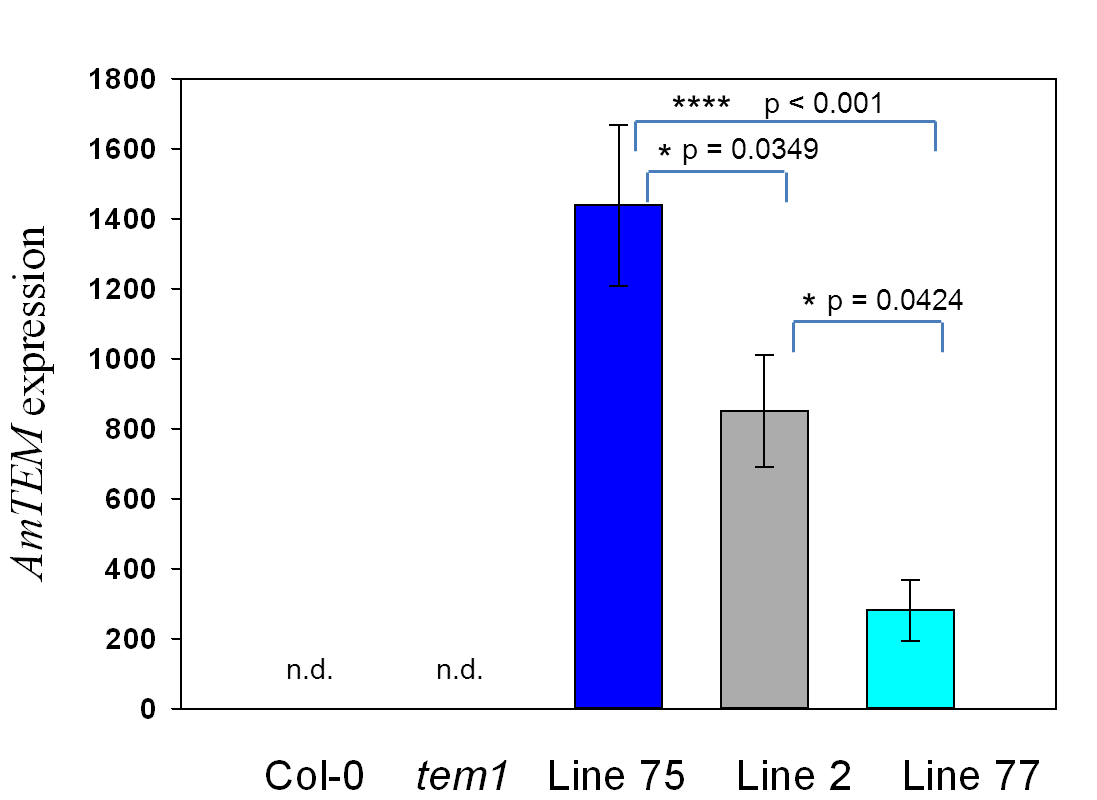


**Supplementary Figure S7. Real-time PCR analysis of *AmTEM* expression in aerial parts of Col-0, *tem1* and T_3_ generation *35S::AmTEM/tem1*  lines grown under LD conditions harvested at ZT15.** *AtTEM1* expression was normalised to *ACTIN2* and *β-TUBULIN* at each timepoint. Expression was analysed in samples at daily intervals from germination over the first 10 days. The average of the first 10 days from germination is presented for each line. The error bars represent the standard error of the normalized relative quantities. n.d. = not detected. Data were analysed by Tukey's multiple comparisons test after two-way ANOVA (p < 0.001) (**** = extremely significant; * = significant).

**Supplementary Figure S8. Flowering time of Col-0 T_1_ generation *35S::AtTEM* lines transformed with *AtTEM1* grown under LD conditions.** Flowering time was assessed by number of rosette leaves when the bolt was 1 cm in length. T_1_ *35S::AtTEM*/Col-0 transgenic lines (grey bars), Col-0 (black bar, n = 68) and *tem1* mutant (white bar, n = 68). Error bars represent the standard error of the mean.


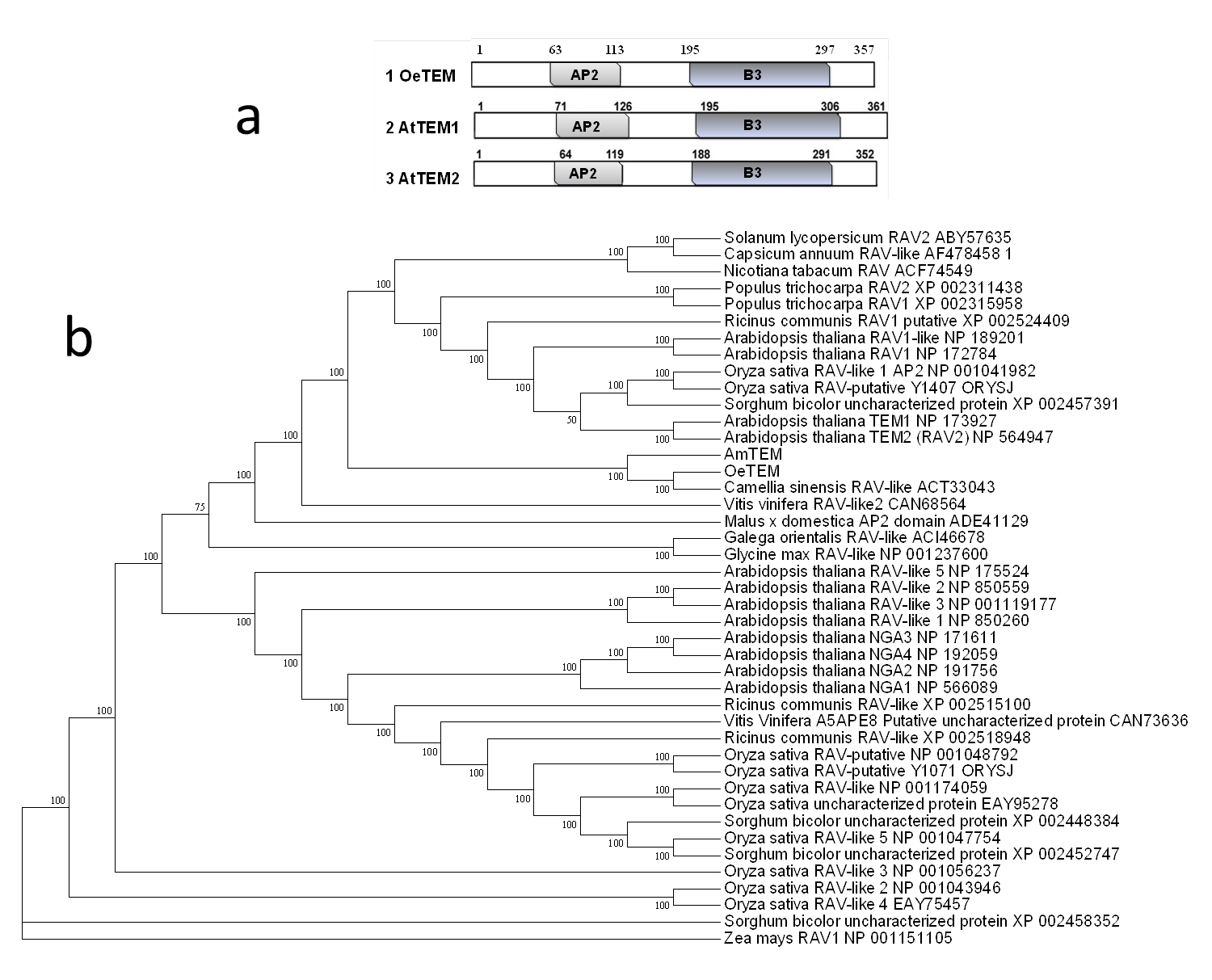


**Supplementary Figure S9. Relationship between OeTEM and RAV sub-family class I members.** a) Comparison of protein domain structure in OeTEM, AtTEM1 and AtTEM2. b) Phylogenetic analysis of AmTEM and OeTEM deduced amino acid sequences and other RAV sub-family class I members homologs. The evolutionary relationship was inferred using the Maximum Parsimony method. The percentage of parsimonious trees in which the associated taxa clustered together are shown next to the branches. Accession numbers are given next to the species name.

**
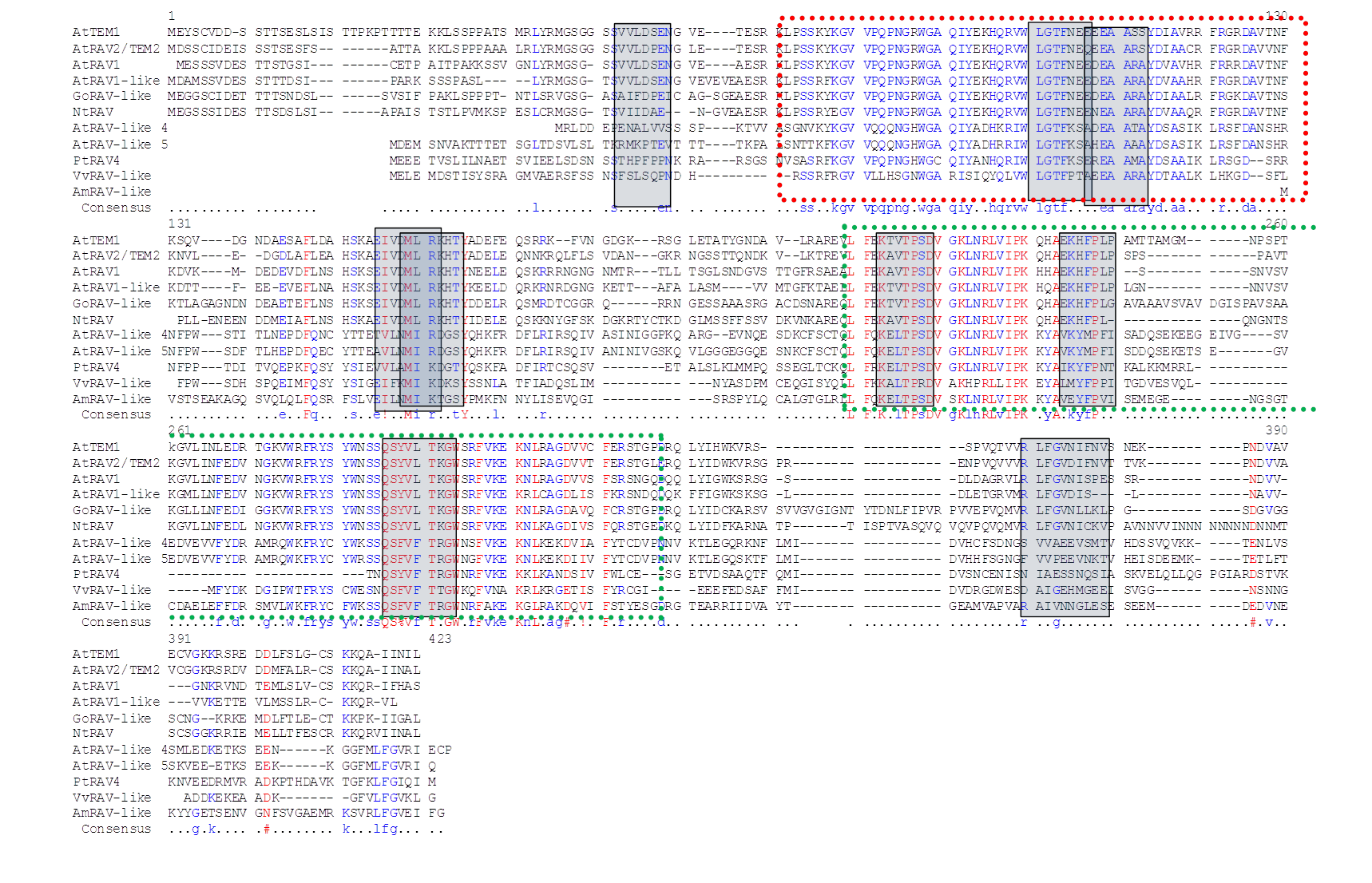
**

**Supplementary Figure S10.** **Regions within RAV and RAV-like protein sequences selected for design of degenerate primers used to isolate *AmTEM*.** AtTEM1 = *Arabidopsis thaliana* TEM1 (NP_173927); AtRAV2/TEM2 = *Arabidopsis thaliana* RAV2/TEM2 (NP_564947); AtRAV1 = *Arabidopsis thaliana* RAV1 (NP_172784); AtRAV1-like = *Arabidopsis thaliana* RAV1-like (NP_189201); AtRAV-like 4 = *Arabidopsis thaliana* RAV-like4 (NP_175483); AtRAV-like 5 = *Arabidopsis thaliana* RAV-like5 (NP_175524); GoRAV-like = *Galega orientalis* RAV-like (ACI46678); NtRAV = *Nicotiana tabacum* RAV (ACF74549); PtRAV4 = *Populus trichocarpa* RAV4 (XP_002308395); VvRAVlike = *Vitis vinifera* RAV-like (XP_002276492); AmRAV-like = *Antirrhinum majus* RAV-like (AJ800976). Black boxes denote where degenerate primers were designed. The red dotted box delimits the AP2 domain, the green dotted box delimits the B3 domain.


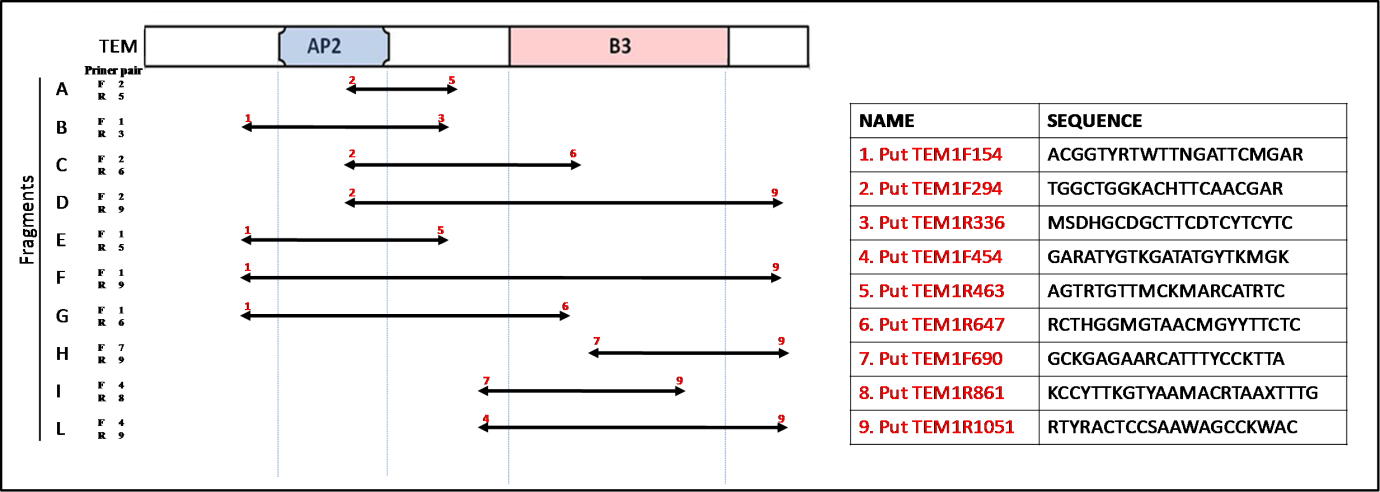


**Supplementary Figure S11. Representation of the annealing position of the 10 combinations of degenerate primers used to isolate *AmTEM* and list of the primer sequences.**


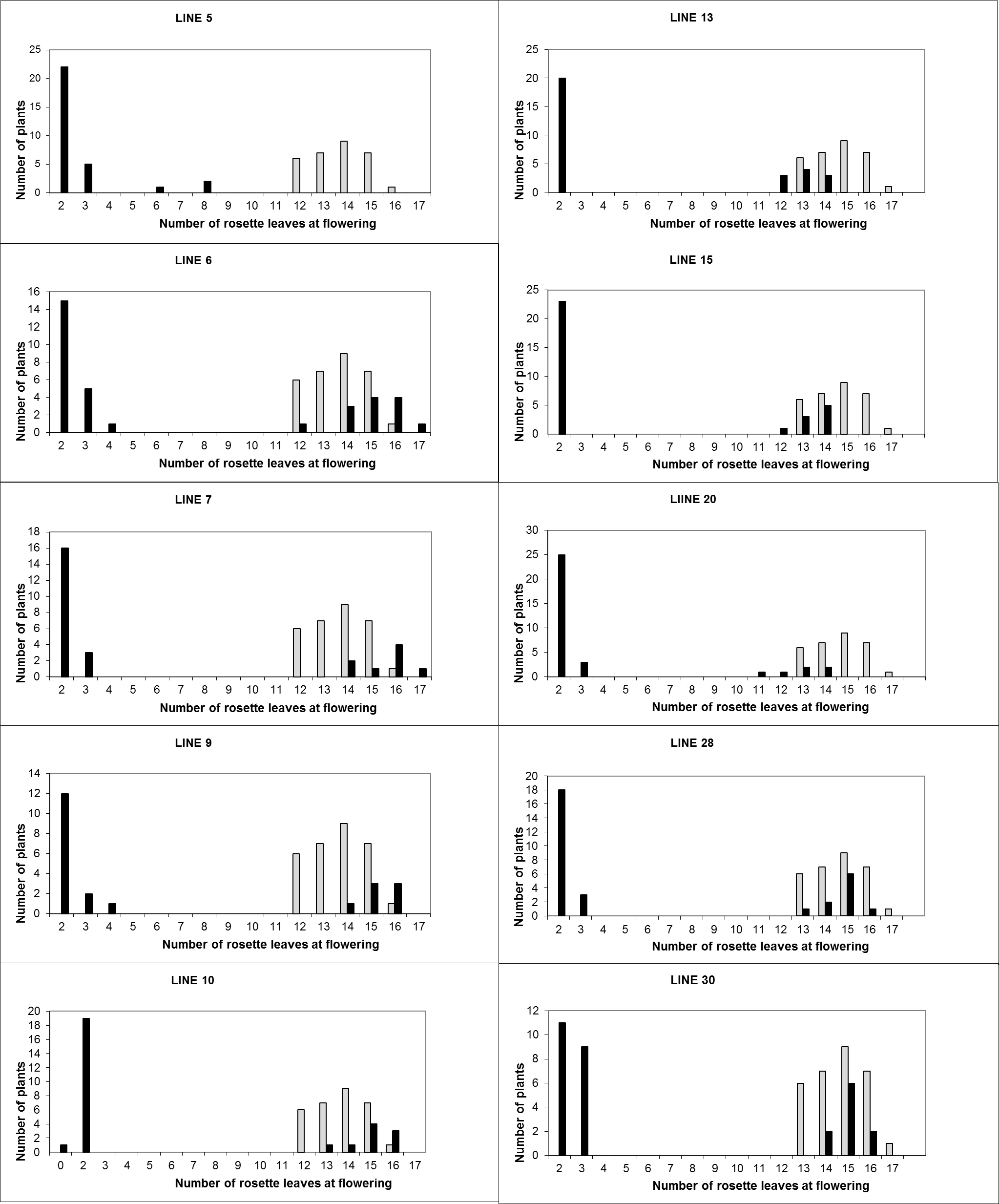


**Supplementary Figure S12. Functional complementation of the *Arabidopsis ft-1* late flowering mutant by *AmFT.*** Flowering times, expressed as number of rosette leaves when the bolt was 1 cm in length, are shown for transgenic T_2_ generation plants from independent T_1_ lines, as indicated (black bars), and non- transformed *ft-1* mutant plants (grey bars) grown under LD conditions. Transgenic lines were engineered to over-express *AmFT* under the control of the *CaMV 35S* promoter. T_2_ generation transgenic plants displayed segregation in flowering times, with several plants flowering earlier than the *ft-1* mutant.

**Supplementary Table S1. Primers used in the study.** In Real-time PCR analysis, 2-step cycling was performed and annealing and extension were carried out at the annealing temperatures shown.

| **Organism and gene (GenBank Acc. No.)** | **Primer name** | **Sequence (5’-3’)** | **Annealing temperature** | **Product size (bp)** | **Final concentration for standard PCR or sequencing** | **Final concentration for Real-time PCR** |
| --- | --- | --- | --- | --- | --- | --- |
|  |  |  |  |  |  |  |
| ***Arabidopsis ACTIN 2* (BE038458)** | AtActin F | TGTCGCCATCCAAGCTGTTCTCT | 63°C | cDNA 85 | 0.5 µM | 0.2 µM |
|  | AtActin R | GTGAGACACACCATCACCAGAAT |  | gDNA 163 | 0.5 µM | 0.2 µM |
| ***Arabidopsis* *β-TUBULIN* (AY040074)** | TUBBY F | TGGCAAGATGAGCACAAAAG | 60°C | 129 |  | 0.4 µm |
|  | TUBBY R | AGACCTCGGGGAGCTATG |  |  |  | 0.4 µM |
| ***Arabidopsis FT* (NM_105222)** | Real-time AtFT F | GGCCTTCTCAGGTTCAAAACA | 55°C | 119 | 0.5 µM | 0.2 µM |
|  | Real-time AtFT R | TCGGAGGTGAGGGTTGCTA |  |  | 0.5 µM | 0.2 µM |
| ***Arabidopsis TEM1* (NM_102367)** | Real-time Tem1 F | CTGGAACAGCAGTCAAAGTTACGTGT | 67°C | 100 |  | 0.2 µM |
|  | Real-time Tem1 R | TGATCTCTCGAAACAAACCACATCAC |  |  |  | 0.2 µM |
| ***Arabidopsis TEM1* (NM_102367)** | AtTEM1-F | ATGGAATACAGCTGTGTAGACGA | 61°C | 1091 | 0.5 µM |  |
|  | AtTEM1091-R | ATTTGTCACAAGATGTTGATAATCG |  |  | 0.5 µM |  |
| ***Arabidopsis TEM1* (NM_102367)** | Seq Attem1 F | ATGGAATACAGCTGTGTAGACGA |  | 1091 | 3.2 μM |  |
|  | Seq Attem1 R | ATTTGTCACAAGATGTTGATAATCG |  |  | 3.2 μM |  |
| ***Arabidopsis TEM2* (NM_105558)** | Real-time Tem2 F | GCCGTTTGCGGTGGAAAGAGAT | 61°C | 104 |  | 0.2 µM |
|  | Real-time Tem2 R | GAAAAGGAAATATGTCACAAAGCAT |  |  |  | 0.2 µM |
| ***Arabidopsis TEM2* (NM_105558)** | Seq Attem2 F | ATGGATTCTAGTTGCATAGACGAG |  | 1073 | 3.2 μM |  |
|  | Seq Attem1 R | GAAAAGGAAATATGTCACAAAGCAT |  |  | 3.2 μM |  |
| ***Arabidopsis CO* (NM_001036810)** | Real-time AtCO F | GAGAAATCGAAGCCGAGGAGCA | 61°C | 80 |  | 0.2 µM |
|  | Real-time AtCO R | TCAGAATGAAGGAACAATCCCATA |  |  |  | 0.2 µM |
| ***Antirrhinum ELONGATION FACTOR α* (AJ805055)** | Ant elf-alpha F | GAGTACCCACCTCTTGGACGTT | 61°C | 92 | 0.5 µM | 0.2 µM |
|  | Ant elf-alpha R | CTGGGGTCTTTCTTCTCAACAC |  |  | 0.5 µM | 0.2 µM |
| ***Antirrhinum ACTIN* (HQ853640)** | QACT ant F | TCAGTGGAGGGTCTACCATGTTTCCTG | 60 °C | 77 |  | 0.2 µM |
|  | QACT ant R | GCTACTGGGAGCCAACGCCGTA |  |  |  | 0.2 µM |
| ***Antirrhinum FT* (AJ803471)** | Ant put FT F | GCCAGAATTTCAACACGAGAGAC | 63°C | 78 | 0.5 µM | 0.2 µM |
|  | Ant put FT R | GGCAATTGAAGTAGACAGCAGCA |  |  | 0.5 µM | 0.2 µM |
| ***Antirrhinum TEM* (JX997989)** | AmTEM1F | ATGGACGGAAGCTGCATAGAC | 63 ºC | 1072 | 0.5 µM |  |
|  | AmTEM1072R | CTAAATGTTACAAAGCATCAATCACC |  |  | 0.5 µM |  |
| ***Antirrhinum TEM* (JX997989)** | Real-time AmTEM F | AATCTGAAAGCGGGCGATGTTGTA | 65°C | 100 |  | 0.2 µM |
|  | Real-time AmTEM R | CCGACCCATTACCATTACTCCTCA |  |  |  | 0.2 µM |
| ***Antirrhinum TEM* (JX997989)** | Seq Amtem F  or CI-Am F | CGGAAACGGAACACGATGACG |  | 167 | 3.2 μM |  |
|  | Seq Amtem R  or CI-Am R | CGACAAAACGAACGGACACGAT |  |  | 3.2 μM |  |
| **Olive *actin1* (AY788899)** | Oe-Actin F | TCCTGAGGTTCTTTACCAGCCTTC | 65°C | 191 | 0.5 µM |  |
|  | Oe-Actin R | CTAGCGCTGTAATTTCCTTGCTCA |  |  | 0.5 µM |  |
| **Olive *TEM* (KC007944)** | OeTEM1F | ATGGATACTAGTTCAATAGGTGAAAGC | 65 ºC | 1074 | 0.5 µM |  |
|  | OeTEM1074R | TTACAAAGCATCAATAACCCTCTGTT |  |  | 0.5 µM |  |
| **Olive *TEM* (KC007944)** | Oe fragment TEM F | CAAAGCTACGTGTTAACAAAAGGAT | 61°C | 312 | 0.5 µM |  |
|  | Oe fragment TEM R | TACAAAGCATCAATAACCCTCTG |  |  | 0.5 µM |  |
| **Olive *TEM* (KC007944)** | SeqOetem F | CAAAGCTACGTGTTAACAAAAGGAT |  | 312 | 3.2 μM |  |
|  | SeqOetem R | TACAAAGCATCAATAACCCTCTG |  |  | 3.2 μM |  |
| **E. coli M13** | M13 F | TGTAAAACGACGGCCAGT |  |  | 3.2 μM |  |
|  | M13 R | GGAAACAGCTATGACCATG |  |  | 3.2 μM |  |

**Supplementary Table S2. Reference gene expression stability and Real-time PCR efficiencies.** Expression stability of reference genes used in *Arabidopsis* and antirrhinum real-time PCR experiments were calculated using geNorm. M = geNorm expression stability value of the reference gene; CV = coefficient of variation of the normalized reference gene relative quantities. Amplification efficiency was calculated for each run. E = efficiency value + 1 (with an E of 2 indicating 100% efficiency).
